# Artificial cloud test confirms volcanic ash detection using infrared spectral imaging

**DOI:** 10.1038/srep25620

**Published:** 2016-05-09

**Authors:** A. J. Prata, F. Dezitter, I. Davies, K. Weber, M. Birnfeld, D. Moriano, C. Bernardo, A. Vogel, G. S. Prata, T. A. Mather, H. E. Thomas, J. Cammas, M. Weber

**Affiliations:** 1 Nicarnica Aviation AS, Vollsveien 9-11, N-1366, Lysaker, Norway; 2 Visiting scientist, Department of Atmospheric, Oceanic and Planetary Physics, University of Oxford, UK; 3 AIRBUS Operations SAS, Toulouse, France; 4 easyJet plc, Luton, UK; 5 Department for Mechanical Engineering, Düsseldorf University of Applied Sciences, Düsseldorf, Germany; 6 AIRBUS SAS, Toulouse, France; 7 Section for Meteorology and Oceanography, Department of Geoscience, University of Oslo, Norway; 8 Atmosphere and Climate Department, Norwegian Institute for Air Research, Kjeller, Norway; 9 Department of Earth Sciences, University of Oxford, UK; 10 Visiting Scientist, School of Earth Sciences, University of Bristol, BS8 1RJ.

## Abstract

Airborne volcanic ash particles are a known hazard to aviation. Currently, there are no means available to detect ash in flight as the particles are too fine (radii < 30 *μ*m) for on-board radar detection and, even in good visibility, ash clouds are difficult or impossible to detect by eye. The economic cost and societal impact of the April/May 2010 Icelandic eruption of Eyjafjallajökull generated renewed interest in finding ways to identify airborne volcanic ash in order to keep airspace open and avoid aircraft groundings. We have designed and built a bi-spectral, fast-sampling, uncooled infrared camera device (AVOID) to examine its ability to detect volcanic ash from commercial jet aircraft at distances of more than 50 km ahead. Here we report results of an experiment conducted over the Atlantic Ocean, off the coast of France, confirming the ability of the device to detect and quantify volcanic ash in an artificial ash cloud created by dispersal of volcanic ash from a second aircraft. A third aircraft was used to measure the ash *in situ* using optical particle counters. The cloud was composed of very fine ash (mean radii ~10 *μ*m) collected from Iceland immediately after the Eyjafjallajökull eruption and had a vertical thickness of ~200 m, a width of ~2 km and length of between 2 and 12 km. Concentrations of ~200 *μ*g m^−3^ were identified by AVOID at distances from ~20 km to ~70 km. For the first time, airborne remote detection of volcanic ash has been successfully demonstrated from a long-range flight test aircraft.

Fine (radii < 30 *μ*m) airborne volcanic ash is composed of irregular shaped minerals and glass components with an SiO_2_ content ranging from 40% to 80%^1^. Copious amounts of ash can be emitted high into the atmosphere from even moderate-sized volcanic eruptions, where the atmospheric circulation can transport it 100 s to 1000 s km, intersecting commercial air routes and presenting a major hazard to aviation^2^,^3^,^4^,^5^. During the April and May 2010 eruption of Eyjafjallajökull, European aviation was grounded for five days causing large economic and societal impacts with estimated losses of US$5bn to the global economy^6^,^7^. Volcanic ash damages jet engines as the glassy components undergo a phase transition between temperatures of 700 and 1100 °C^8^, becoming plastic in the hot sections of the turbine^9^. Upon liquefying, the glass sticks to metallic surfaces, reducing engine efficiency and potentially clogging inlets/outlets. Although a reduction in engine temperature will result in the glass solidifying, fracturing and clearing the metal surfaces, this is still an extremely undesirable occurrence. Volcanic ash particles can also migrate through the engine without liquefying, blocking inlets/outlets and abrading engine components. Other deleterious effects that have been identified include: clogging the air bleed filter system with consequent loss of pressurisation, short circuit and intermittent failure of electronic components, and obstructing the pitot-static system leading to unreliable speed indications^2^,^3^. Some, or all, of these effects can lead to an in-service event causing engine damage or engine power loss and potential loss of aircraft, passengers and crew. At least two incidents^9^,^10^ due to volcanic ash encounters, have led to loss of power to all engines but, as yet, no air crashes have been attributed to a volcanic ash encounter. General aviation has been concerned with the hazard from airborne ash for at least 30 years, and following the June 1991 eruption of Pinatubo, Philippines, the International Civil Aviation Organisation (ICAO) established nine Volcanic Ash Advisory Centres (VAACs), located within meteorological watch offices to guide global aviation. These centres utilize data from ground-observers, volcano observatories, pilot reports, satellite, ground and airborne instruments (where available) and dispersion model forecasts to provide volcanic ash advisories for civil aviation^11^. Ash clouds are difficult or impossible to detect by eye^12^. The unpredictability of volcanic eruptions and the lack of information on the quantity, size distribution, composition, shape and height distribution of ash emitted to the atmosphere during eruptions mean that quantitative information on ash concentrations is uncertain^12^. The significant hazard that ash presents to aviation coupled with poor knowledge of ash concentrations has led to a very cautious approach to the problem, with significant disruption to air traffic during ash incursions on airspace.

An airborne, fast sampling (~1 Hz), dual-wavelength (spectral) uncooled infrared imaging camera system has been developed to test its ability to detect and quantify ash in the atmosphere from distances of up to 100 km. The Airborne Volcanic Object Imaging Detector (AVOID) is designed for use at typical cruise altitudes (34,000–42,000 ft or 10–16 km) and speeds (~900 km hr^−1^) and views the atmosphere ahead of the aircraft. The system utilises two wavelength regions to detect the signature of SiO_2_ in the ash particles, and permit discrimination from other meteorological clouds of water vapour, water droplets and ice crystals. The basis of the method has been described in several publications^13^,^14^,^15^,^16^ and a similar detection scheme using satellite measurements is employed by the VAACs. A prototype system was tested using a light aircraft at Mt Etna and Stromboli volcanoes, Italy during November 2011, where it was taken to 12,000 ft altitude and eruption clouds from both volcanoes were imaged. In July 2012 the system was pod mounted on the side of the fuselage of an AIRBUS A340 flight-test aircraft. During these tests, AVOID was flown at speeds of up to 960 km hr^−1^ and reached altitudes of 38,000 ft. Imagery of meteorological clouds was acquired at 1 Hz frequency from the forward looking cameras and an algorithm used to determine the nature of the clouds. Specifically, the system was being tested to identify water and ice clouds, and perform sensitivity analyses to quantify detection limits based on the noise equivalent temperature differences (NEΔTs of 50–200 mK) of the uncooled microbolometers. The ability to identify thin layers at great distances (~100 km) and to identify clouds at night was verified. A false detection rate (incorrectly identifying pixels as ash) of 7% was determined in the ash-free atmosphere. This rate can be easily reduced to 0% by selecting different thresholds in the algorithm, however without a test in an ash environment, altering thresholds may reduce positive detection of ash. Also, during these tests, the system was flown on a long traverse towards the Canary Islands, where a boundary layer windblown desert sand was correctly identified using the same algorithm that exploits the ‘reverse absorption’ effect^13^ due to SiO_2_ at the two AVOID wavelengths. Desert sand presents a signature that is similar to volcanic ash in the AVOID system, because of its high SiO_2_ content. While AVOID cannot distinguish airborne ash from windblown sand, both are hazardous to aviation and windblown sand is mostly found at much lower altitudes than volcanic ash.

Following these successful trials, it was decided that the system should be tested at an ash producing volcano. However, the logistical constraints on the A340 aircraft and availability of reliable ash producing eruptions with columns reaching heights of 10,000 ft or higher made it extremely difficult to plan and conduct such an experiment. Instead, a desk study showed that it was feasible to generate a small ash cloud in the atmosphere at a predetermined place and time without compromising air safety or affecting the environment. Approximately 1000 kg of fine ash with a size distribution having a mean radius of 10 *μ*m and geometric standard deviation *σ* = 1.8 was injected into the atmosphere at an altitude of ~12,000 ft from an AIRBUS A400M flight-test aircraft executing an upward spiral flight path, over a small region of sea in the Bay of Biscay (Fig. 1). The ash formed a thin layer ~200 m deep and quickly dispersed horizontally to form an ash cloud approximately 2 km wide and 12 km long, but was not visible to the eye or to visible wavelength cameras (Fig. 1a). Within 30 minutes of the ash cloud layer forming, the A340 aircraft carrying the AVOID instrument flew towards it from approximately 80 km distance at an altitude of 15,000 ft (Fig. 1b, flight run 1). The aircraft turned at approximately 20 km from the location of the ash layer. Three further approaches were made toward the ash layer from altitudes of 10,000 ft (flight run 2), 5,000 ft (3) and 5,000 ft (4). The purpose of these flight runs was to image the ash from above, along the limb and from below. During the injection of the ash into the atmosphere, the ash layer was initially visible to the naked eye, but within a few minutes it was no longer possible to identify the layer. In order to obtain verification of the AVOID measurements of the ash layer, a small aircraft (a Diamond DA42 MPP) equipped with optical particle counters (OPCs) flew into the ash layer and made *in situ* measurements of the ash.

Ash was identified by the AVOID system on three of the four flight runs; no ash was detected from 15,000 ft because the ash layer was too low to be within the field of view of the cameras. On flight runs 3 and 4 (5,000 ft) it was possible for AVOID to identify the small aircraft flying within the ash layer. AVOID first reliably identified the layer from a distance of ~67 km. At that distance individual camera pixels measure ~10 × 10 m^2^, giving approximately 20 vertical pixels for the layer ~200 m deep. Mass loadings along the line of sight of the instrument were calculated (see Methods) and ranged from 0.1 g m^−2^ to 1.6 g m^−2^. These convert to concentrations of ~200–3200 *μ*g m^−3^, using an ash cloud width of ~2 km, as measured by the DA42. Figure 2, Panel (a) shows the times when ash was detected during flight run 3. Detections are only possible when the field of view of the instrument, indicated by the hatched region, intersects the location of the ash cloud centred at ~11,000 ft. Because the attitude of the aircraft is changing, the field of view coverage changes and corrections must be applied to the mass loading retrieval to account for optical path changes. Panel (b) shows a single processed image frame from AVOID (movie loops for all four runs are provided in the Supplementry Materials), indicating the location and mass loading of the ash layer. The DA42 *in situ* measurements, shown as solid circles, have been collocated onto the image and their size is proportional to the mass concentrations measured by the OPC. Panel (c) shows a summary of detections from three flight tracks at ranges from 10–65 km. Assuming a homogeneous ash layer ~200 m deep by ~2 km wide and ~12 km long (see Methods), we can calculate the theoretical detection probability for the imaging device at varying distances from the cloud. This can be compared with the actual detection, expressed as a percentage of the total number of pixels within the field of view of the cameras. The results indicate very good detection (compared to the theoretical curve) for distances from ~25 to 60 km. The theoretical detection threshold takes no account of inhomogeneities in the ash cloud or changes in the pitch of the instrument that affect the path length and hence mass loading sensitivity. As the aircraft approached within 20 km of the cloud, the imager began to observe the cloud from increasingly large viewing zenith angles, reducing the path length and also observing through the centre of the ash cloud, which contained concentrations below the limit of detection of AVOID, <~100 *μ*g m^−3^ for ash cloud thicknesses of ~2000 m. Hence at distances of ~20 km from the cloud ash detection was problematic.

The vertical depth of the ash layer was verified by descending and ascending the DA42 until negligible particles were counted. The AVOID measurements suggest a descent of the ash layer of ~0.3 m s^−1^ (Fig. 3a), broadly consistent with terminal velocities for spherical particles of density ~2600 kg m^−3^. The distribution of mass determined from the OPC measurements indicates two peaks: a background peak near 70 *μ*g m^−3^ and a second peak near 400 *μ*g m^−3^, associated with the ash cloud (Fig. 3b). These measurements (Fig. 3c) also show that the layer was not more than ~300 m deep. The OPCs measured ash concentrations between 100 *μ*g m^−3^ to 6000 *μ*g m^−3^ with a very high degree of heterogeneity in the cloud (Fig. 3c). The smaller range of concentrations (~100 to ~600 *μ*g m^−3^) measured by AVOID compared to the OPCs may be a consequence of the averaging process of the imager and is also due to the assumption of a uniform width of the ash cloud.

The sensitivity of the AVOID system depends on the mean effective radius of the ash size distribution and the composition of the ash. We measured the ash size distribution before and after the experiment and found no discernible difference in the mean radius (~10 *μ*m). The composition of the ash was measured before the experiment; the sample had a complex structure (Fig. 4a) and contained a significant glassy proportion (Fig. 4b). The composition was dominated by SiO_2_ (Fig. 4c).

The identification of an ash layer embedded within an atmosphere containing meteorological clouds from distances of up to ~60 km, provides a warning time of 4 minutes for an aircraft travelling at 900 km hr^−1^. This is sufficient time to make a gradual change in course direction and avoid intersecting the ash layer. Using the Caliop (Cloud-Aerosol Lidar with Orthogonal Polarisation) lidar and AIRS (Atmospheric Infrared Sounder) satellite instrument data, it was found that during the Eyjafjallajökull, Chaitén (May 2008), Sarychev Peak (June, 2009), Puyehue-Cordón Caulle (June, 2011) and Calbuco (April 2015) eruptions the ash layers were thin; ranging from 500 m to 3000 m deep^17^,^18^. Ground-based^19^,^20^ and airborne^21^,^22^ lidar measurements and modelling^23^,^24^ of dispersing ash layers also support this finding.

Near real-time satellite detection of ash clouds is currently used by VAACs and dispersion and transport models are used to forecast their concentration and movement. However, dispersing ash clouds forming in thin layers (<~2000 m) may not be detected by satellite instruments, and may not be vertically resolved by current dispersion models. While satellite data and model forecasts can provide strategic information for airlines to plan their operations^5^, uncertainties in eruption source parameters coupled with uncertainties in forecast wind measurements lead to errors in the forecast ash cloud location^11^. Furthermore, the recent eruption of Kelut volcano, Indonesia on 13 February 2014 demonstrated that even with good information on the location and timing of the eruption, it did not prevent a commercial aircraft from encountering an ash layer and damaging its engines. In this case, the encounter occurred in low light, in an ash layer that was hidden from the satellites’ view by a larger ash umbrella cloud. Modelling^25^ showed that the aircraft likely encountered this cloud for several minutes and that ash concentrations ranged from 2000 *μ*g m^−3^ to 9000 *μ*g m^−3^. An airborne detection system would have alerted the aircraft and allowed a timely and safe routing option for the aircraft to avoid an encounter with the ash cloud.

The results from this experiment successfully demonstrate remote detection of ash clouds using thermal imaging cameras. An earlier experiment in skies containing no ash gave a false detection rate of ~7%, but with some fine tuning of the thresholds used in the algorithm, improvements in the sensitivity of uncooled bolometric detectors and judicious choice of the bandpasses used for filtering the radiation this rate can be reduced to below 3%. This experiment used a very small ash cloud (<10% of pixels contained ash) and when compared with the expected theoretical signal, we have shown that if >1% of pixels contain ash then a positive detection can be made. It is stressed however, that this experiment was conducted at the relatively low altitude of ~11,000 ft; at higher altitudes atmospheric conditions will be different and the bandpasses for the two cameras will require optimisation. The composition of the ash is also an important factor affecting the sensitivity of detection in the thermal infrared, since the silicate content and ‘glassiness’ of the ash determine the variation of extinction of radiation as a function of wavelength^26^. The mineral composition of the Eyjafjallajökull sample used in this experiment was measured and had a high SiO_2_ content (Fig. 4c). Some of the particles were highly irregular, possibly due to interaction with water during the fragmentation process (Fig. 4a,b). Radiative transfer modelling is currently underway to determine the optimal filter positions to ensure maximum ash detection for a range of ash compositions and shapes for thin ash layers in the altitude range of 5,000 ft to 45,000 ft, while also minimising the false detection rate.

## Methods

### Volcanic ash

**Figure 4:**
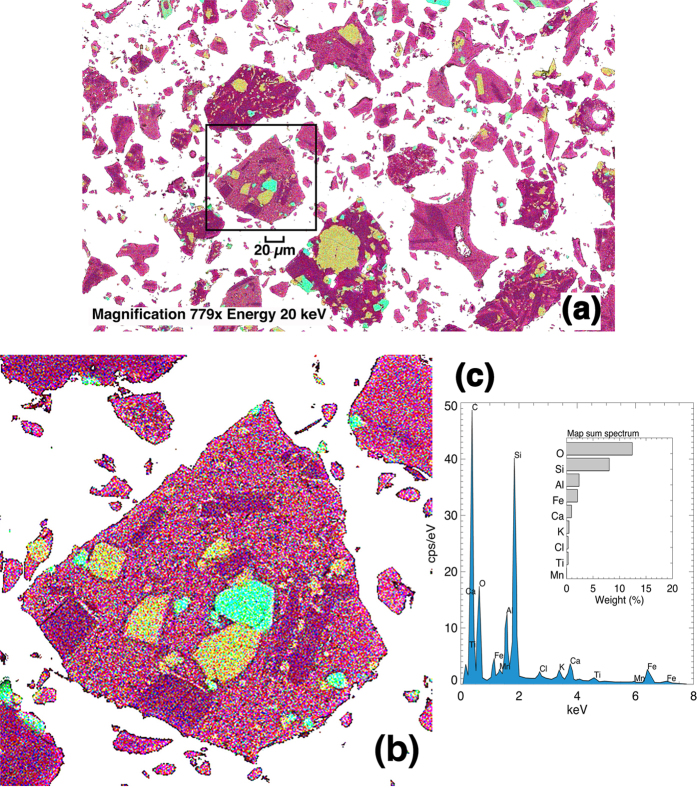
(**a**) Energy Dispersive X-ray Spectroscopic (EDS) elemental map of the Eyjafjallajökull ash sample. The influence of water during fragmentation has resulted in the blocky and irregular shapes of the particles. (**b**) A magnified portion of the image showing the highly complex mixture of elements contained in the various crystalline and glassy structures. The colours represent counts of each of the following elements in order of abundance: red = Si, blue = Al, green = Fe, yellow = Ca, magenta = K, and cyan = Ti. The crystals dominated by Ca (yellow) are Ca-rich pyroxene (augite), crystals dominated by Fe and Ti (green and cyan) are ilmenite, crystals highlighted by a lack of Ca (yellow) and presence of Al (blue) and K (magenta) are most likely a Al-K-rich plagioclase (orthoclase). These crystals are set in an amorphous groundmass of glass. (**c**) Spectrum of counts per second per electron volt. These energies allow identification of elements and their semi-quantitative proportions.

In March 2013 1000 kg of fine ash was sourced from deposits of Eyjafjallajökull ash collected by the University of Iceland shortly after the 15 April, 2010 eruption. The ash was transported to Toulouse, France and stored in 25 kg containers. The ash was milled to remove larger particles, more representative of ash transported over long distances (>~1000 km). Samples of this ash were analysed at the University of Oxford to provide composition (Fig. 4) and size distribution information.

### AVOID installation

The AVOID system was deployed in an aerodynamic pod, mounted on the port side of an AIRBUS A340 flight-test aircraft specially equipped to conduct experimental trials. The pod was secured onto metal plates replacing two forward windows and attached using struts. The head of the AVOID system consists of two fast sampling (~50 Hz) infrared cameras with F/1.2 optics and interference filters placed behind the lenses. Dry air is passed through the pod, but no temperature stabilisation (heater) was deployed because previous tests had shown that the pod remains warm (> −10 °C) due to heat from the cameras and associated electronics. The pod was lined internally with aluminium foil insulation pads and its humidity and temperature were monitored continuously. The cameras were protected from windblown material damage using a hard carbon coated Germanium window of 5 mm thickness. Prior to flying, the system was pre-calibrated in the laboratory using a standard blackbody and an environmental chamber to simulate the expected cold temperatures during flight at high altitude. All of the important instrumental functions were monitored continuously during the flights, including the temperature of the optics (in four locations), the focal plane array (FPA) temperature, the internal pod temperature and humidity and the accelerations of the pod during flight using pressure transducers. Aircraft attitude data were also acquired by the AVOID system, but more accurate data from the A340 avionics system were used in the post analysis. Data were received in real time onto a control computer housed inside the aircraft and displayed onto a screen. Only an indication of the ash layer was provided during the flights; detailed analysis and corrections for aircraft attitude were applied in a post analysis. As it was impossible to visually sight the ash layer, the first indications of it were made using the AVOID data displayed on the system on the aircraft in real time. However, proper identification and analysis, including conversion of the brightness temperature measurements to mass loadings required use of a radiative transfer model and a microphysical ash model (see Radiative Transfer in Methods).

### Ash deposition experiment

The generation of the ash cloud was conducted from an AIRBUS A400M flight-test aircraft. Two specially designed nozzles were used with hosepipes feeding ash from 25 kg containers mounted on racks inside the aircraft. Operators moved the hosepipes to a fresh ash container as each one was emptied into the atmosphere. The ash was forced from inside the aircraft to the outside by making use of the differential pressure. Flow rates of ~0.8 kg s^−1^ were achieved using two nozzles, and a total of 975 kg was injected into the atmosphere in 20 minutes. The ash was dispersed as the A400M climbed vertically in a tight spiral track, leaving a cloud that resembled a torus with an internal radius of ~1000 m. By the end of the measurement period (approximately 40 minutes from the start) the ash layer generated was ~200 m deep, ~2 km wide and ~12 km long, with a “hole” at its centre. Integrating over this volume the total mass estimated by AVOID is ~900 kg, while the mass estimated by integrating the OPC measurements is ~950 kg. Both estimates compare favourably with ~975 kg that was released from the A400M.

### Radiative transfer

We consider a plane-parallel cloud consisting of a distribution of spherical particles within a scattering layer, and solve the following radiative transfer equation (RTE)^27^:


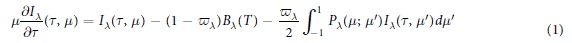


where *I*_*λ*_(*τ*, *μ*) is the radiance measured by the sensor at wavelength *λ* in the direction *μ*. *τ* is the optical depth, *μ* is the cosine of the zenith angle, *ϖ*_*λ*_ is the single scattering albedo and *P*_*λ*_ is the axially-symmetric phase function. The cloud layer has a geometrical depth *s* in the direction *μ*. This equation can be solved numerically using appropriate boundary conditions that depend on the viewing geometry, and the radiation incident at the front and back surfaces of the ash cloud^28^,^29^. The parameters *ϖ*_*λ*_, *P*_*λ*_ and *β*_*λ*_, depend on the complex refractive index of the ash particles, the size distribution and their shape. We use a Mie scattering program^29^ to calculate these parameters as a function of *λ*, using a log-normal size distribution fitted to our measurements, assuming spherical particles and refractive index data for Eyjafjallajökull ash. Once these parameters have been calculated for the bandpasses used by the AVOID cameras, the mass loading in the direction *μ* can be found from:


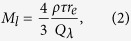


where *ρ* is the density of the ash, *r*_*e*_ is the effective particle radius, 
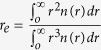
, *Q*_*λ*_ is the non-dimensional Mie extinction coefficient (wavelength dependent), and *n*(*r*) is the size distribution. The concentration *C*, (in kg m^−3^) is the mass loading divided by the the total path *L* 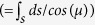, traversed by the radiation through the cloud. For trachyandesite ash (*ρ* = 2.6 × 10^−3^ kg m^−3^) in a homogeneous cloud of 100 *μ*g m^−3^ concentration, *r*_*e*_ ~ 10 *μ*m, *Q* ~ 2.5, the vertical optical depth per kilometre *τ*/*s* ≈ 10^−2^/cos *μ*. It can be seen that AVOID is sensitive to thin ash layers where *L* → 0 because of the limb viewing capability.

### *In situ* particle measurements

The optical particle counter (OPC) was equipped in an aerodynamic measurement pod configuration for a Diamond Airborne Sensing aircraft (DA42-MPP). The measurement pod was installed on the nose of the aircraft to avoid turbulence or other aerodynamic unfavourable effects on the particle inlet. The OPC was connected to an isokinetic straight metal sample inlet tube that was constantly heated up to 10 °C. The OPC, a modified Grimm SkyOPC Model 1.129, was pre-calibrated for volcanic ash particles and consists of a focused laser beam with a fixed wavelength (*λ* = 655 nm) and an optical measurement cell. OPCs combine the principles of light scattering of small particles with single particle counting. By the interaction of particles with this laser band, a light pulse travels in a specific direction that depends on the size of the particle. A pin diode detects the scattered radiation signal from each particle and the downstream pulse height analyser classifies the scattered light pulse into a size distribution (0.25 to 40 *μ*m), based on the optical and physical properties of ash particles. This particle size distribution is converted into a mass concentration of total suspended particle mass (TSP) by assuming size dependent material densities that were derived by the manufacturer. The instrument operates at a volumetric flow rate of 1.2 l/min and a time resolution of 1 s. The accuracy of these airborne particle measurements systems is ±10% and is related to the measurement principle and the saturation level of up to 2.0 × 10^3^ particles/cm^3^.

## Additional Information

**How to cite this article**: Prata, A. J. *et al.* Artificial cloud test confirms volcanic ash detection using infrared spectral imaging. *Sci. Rep.* **6**, 25620; doi: 10.1038/srep25620 (2016).

## Supplementary Material

Supplementary Information

Supplementary Information

Supplementary Information

Supplementary Information

Supplementary Information

Supplementary Information

## Figures and Tables

**Figure 1:**
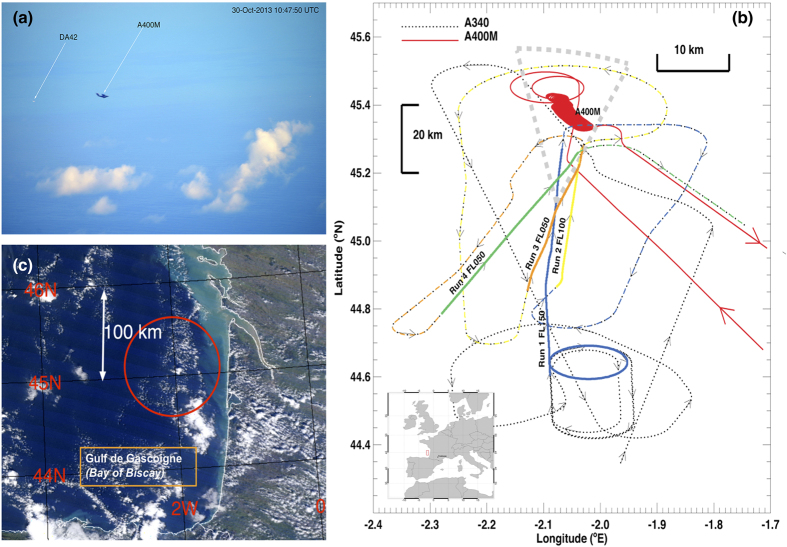
(**a**) Photograph taken from the A340 showing the A400M dispensing ash into the atmosphere. The DA42 can also be seen sampling part of the dispersing ash layer. (**b**) Locations of the A340 aircraft runs. The track of the A400M, dispensing the ash, is shown as a red solid line; the A340 tracks are shown as broken lines and colour coded as follows: blue = run 1, flight level 150 (15,000 ft); yellow = run 2, flight level 100 (10,000 ft); orange = run 3, flight level 050 (5,000 ft); green = run 4, flight level 050. The arrows indicate the direction of travel of the aircraft. The grey-coloured thick-dashed lines show the approximate horizontal field-of-view of the AVOID cameras. Inset map shows the geographic location of the experiment. (**c**) Satellite image (MODIS/Aqua: http://rapidfire.sci.gsfc.nasa.gov/cgi-bin/imagery/realtime.cgi?date=2013303) acquired ~30 minutes after the ash was first inserted into the atmosphere. The ash layer was not detectable at the spatial resolution (250 m) of the visible channels of the MODIS instrument. The map was drawn using the IDL v8.2 software package (www.exelisvis.com). The MODIS data are courtesy of NASA/GSFC and processed using IDL v8.2.

**Figure 2:**
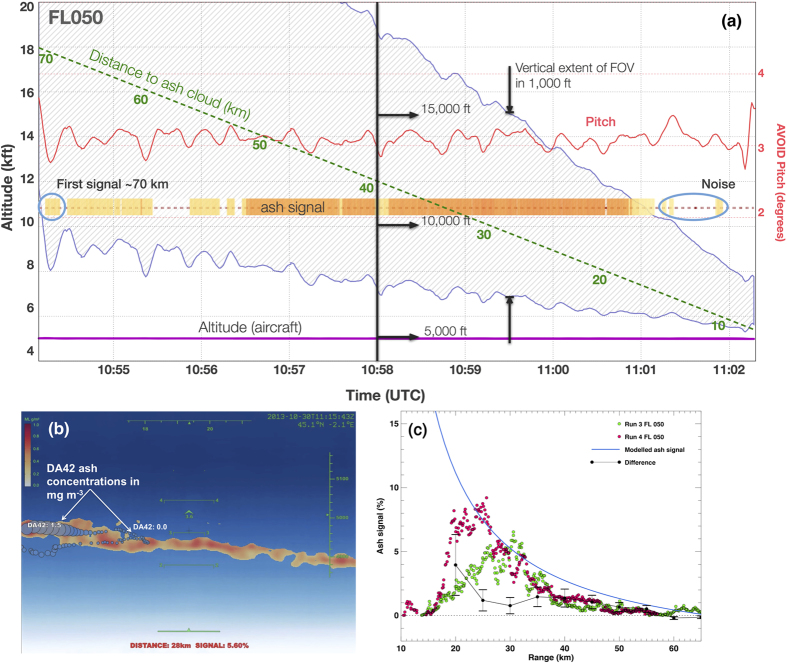
(**a**) Ash signal observed by the dual infrared camera imaging system, AVOID on board the A340 test aircraft from 5000 ft (FL050) viewing the ash cloud at ~11,000 ft from distances of ~70 km. The vertical field-of-view of the system is shown as the hatched coloured region, the A340 altitude is constant at 5000 ft, while the pitch of the instrument (shown in red) undergoes small changes. The total time interval is ~8 minutes. The ash signal is shown in shades of yellow (weaker signal) and orange (stronger signal). The solid vertical line at 10:58 UT corresponds to the time when the DA42 was sampling inside the ash cloud and a vertical profile at this time is shown in Fig. 3(a). (**b**) A single AVOID image frame showing the ash detection signal (yellow/orange) and coincident ash concentrations measured by the DA42 (filled circles). The background shows brightness temperatures from the reference channel in shades of blue to white (cold to warm). (**c**) Ash signal (in %; filled circles) as a function of distance (km) from the ash cloud, shown for two flight runs. The signal is defined as the ratio of the number of pixels identified as ash to the total number of pixels, expressed as a percentage. The solid blue curve shows a theoretical detection limit based on the geometry of the cloud, the pixel resolution (distance dependent) and perfect detection of ash regardless of the amount. The difference between the measured signal and the theoretical estimates is shown every 5 km (black circles) with the standard deviation over 5 km also shown. Beyond 25 km the difference is at or below 1%.

**Figure 3:**
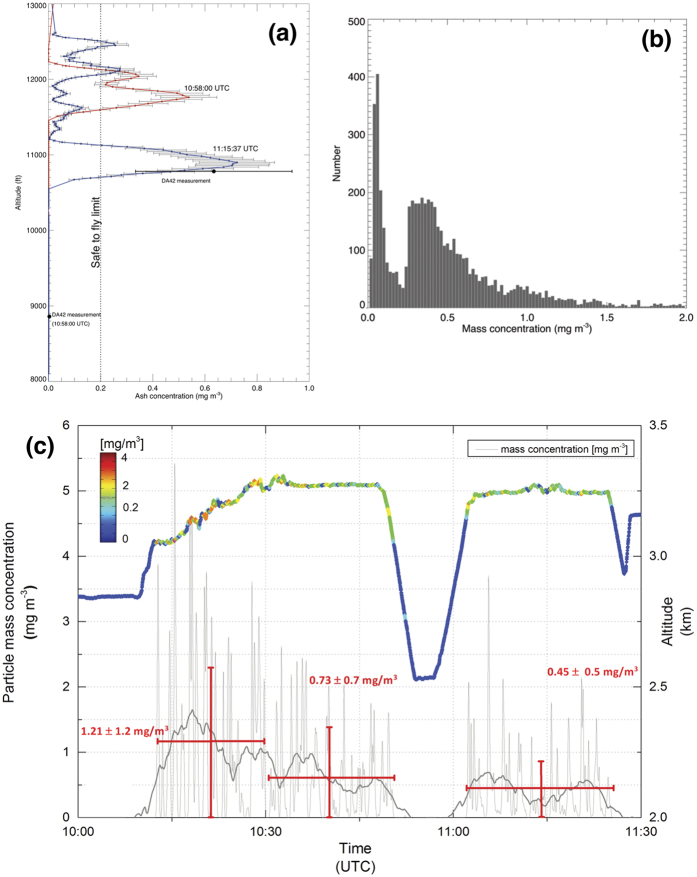
(**a**) AVOID measurements at two times: 10:58:00 UTC when the DA42 aircraft had descended below the ash layer, and 11:15:37 UTC when the DA42 was flying within the ash layer. The ash layer was thin (<300 m deep) and multilayered. (**b**) Histogram of the mass concentration measured by the OPC showing a peak at around ~70 *μ*g m^−3^ representing the background particulate concentration and a broader peak between 250 and 450 *μ*g m^−3^ representing the ash layer concentration. (**c**) *In situ* OPC measurements of the airborne ash made during the experiment. The upper line shows the altitude of the DA42 aircraft, with colours representing the particle mass concentration. Particle mass concentration (mg m^−3^) is plotted as a function of time for a period when the AVOID system was viewing the ash layer. Between 10:50 and 11:00 UTC the aircraft descended until negligible particles could be counted, and the layer depth at this time is estimated to be ~280 m. Over a period of ~1 hour the mean concentration dropped from 1200 to 450 *μ*g m^−3^.
