# Supplementary material for: Artificial cloud test confirms volcanic ash detection using infrared spectral imaging
Source: Sci Rep. 2016 May 9;6:25620. doi: 10.1038/srep25620 (PMC4860601; doi:10.1038/srep25620)
Supplement: Supplementary Information [file srep25620-s1.pdf]

Artificial cloud test confirms volcanic ash detection using infrared spectral imaging–Supplementary material

A. J. Prata[1,7], F. Dezitter[2], I. Davies[3], K. Weber[4], M. Birnfeld[5], D. Moriano[1], C. Bernardo[1], A. Vogel[4,8,9], G. S. Prata[6], T. A. Mather[6], H. E. Thomas[1,10], J. Cammas[5], M. Weber[2]

[1]*Nicarnica Aviation AS, Gunnar Randers vei 24, Kjeller, Norway.*

[2]*AIRBUS Operations SAS, Toulouse, France.*

[3]*easyJet plc, Luton, UK.*

[4]*Department for Mechanical Engineering, Düsseldorf University of Applied Sciences, Düsseldorf, Germany.*

[5]*AIRBUS SAS, Toulouse, France.*

[6]*Department of Earth Sciences, University of Oxford, UK.*

[7]*Visiting scientist, Department of Atmospheric, Oceanic and Planetary Physics, University of Oxford, UK.*

[8]*Section for Meteorology and Oceanography, Department of Geoscience, University of Oslo, Norway.*

[9]*Atmosphere and Climate Department, Norwegian Institute for Air Research, Kjeller, Norway.*

[10]*Visiting Scientist, Department of Earth Sciences, University of Bristol. BS8 1RJ.*

Supplementary Material

Movie files from all four runs are available as supplementary materials. All videos were created using a custom Python script that reads the FITS files and generates a JPEG image at $\sim$1Hz frequency. The images are then passed on to an open source tool called ffmpeg that encodes them in the mp4 format. These were converted to mov format files using Apple Quicktime. All processed data are available as digital data in FITS file format with calibrated brightness temperatures, instrument parameters, aircraft attitude information, GPS location and camera instrument data. These data can be requested from David Moriano through email at dm@nicarnicaaviation.com.

Ash Tanker Test 30 October 2013 - Run 1 FL150.mp4 AVOID dual band imagery showing broadband temperatures (Kelvins; white=warm, blue=cold) of the sky ahead of the A340. Time resolution is approximately 1 s. FL150=flight altitude of 15,000 ft. No ash signal (indicated in shades of yellow/orange/red) was detected on this run.

Ash Tanker Test 30 October 2013 - Run 2 FL100.mp4 AVOID dual band imagery showing broadband temperatures (Kelvins; white=warm, blue=cold) of the sky ahead of the A340. Time resolution is approximately 1 s. FL150=flight altitude of 10,000 ft. A seconds of data (10:38:51-10:38:53Z) indicated an ash signal at the extremity of the camera's field of view.

Ash Tanker Test 30 October 2013 - Run 3 FL050.mp4 AVOID dual band imagery showing broadband temperatures (Kelvins; white=warm, blue=cold) of the sky ahead of the A340. Time

resolution is approximately 1 s. FL050=flight altitude of 5,000 ft. A noisy ash signal is first observed at 10:54:23Z, when the A340 was approximately 68 km from the ash layer. The ash signal becomes increasingly coherent as the aircraft approaches, and a steady signal is observed from 50 km. Note that the circular, white coloured (warm) feature moving across the image is the DA42 aircraft.

Ash Tanker Test 30 October 2013 - Run 4 FL050.mp4 AVOID dual band imagery showing broadband temperatures (Kelvins; white=warm, blue=cold) of the sky ahead of the A340. Time resolution is approximately 1s. FL050=flight altitude of 5,000 ft. A repeat run at 5,000 ft also showing coherent detection from distances of $\sim$50 km.

Ash Tanker Test 30 October 2013 - DA42.mp4 AVOID dual band imagery showing broadband temperatures (Kelvins; white=warm, blue=cold) of the sky ahead of the A340 for run 4, with ash mass loadings overlaid and collocated with the position of the DA42. The circles indicate positions where mass concentrations were measured by the OPCs and their size is proportional to the mass concentration.
